# The ACE Index: mapping childhood adversity in England

**DOI:** 10.1093/pubmed/fdz158

**Published:** 2019-12-27

**Authors:** Dan Lewer, Emma King, Glen Bramley, Suzanne Fitzpatrick, Morag C Treanor, Nick Maguire, Miriam Bullock, Andrew Hayward, Al Story

**Affiliations:** 1 UCL Collaborative Centre for Inclusion Health, Institute of Epidemiology and Health Care UCL, 1-19 Torrington Place, London, WC1E 7HB, UK; 2 Institute for Social Policy, Housing, Equalities Research; Heriot-Watt University, Edinburgh, EH14 4AS, UK; 3 Psychology, University of Southampton, University Road, Southampton SO17 1BJ, UK; 4 Find&Treat, University College London Hospitals NHS Foundation Trust, London NW1 2PG, UK

**Keywords:** children, public health, social determinants

## Abstract

**Background:**

Studies of adults show that adverse childhood experiences (ACEs) are associated with health and social problems and are more common among people living in deprived areas. However, there is limited information about the geographical pattern of contemporary ACEs.

**Methods:**

We used data from the police, social services, schools and vital statistics in England to calculate population rates of events that represent childhood adversity. We constructed an ‘ACE Index’ that summarizes the relative frequency of ACEs at local authority level, informed by the methods of the Index of Multiple Deprivation. We explored associations between the ACE Index and local characteristics in cross-sectional ecological analysis.

**Results:**

The ACE Index was strongly associated with the proportion of children that live in income-deprived households (child poverty). In addition, the ACE Index was independently associated with higher population density and was higher in certain regions, particularly the north-east.

**Conclusions:**

The association between ACEs and child poverty provides evidence of a process in which deprivation increases the risk of adverse experiences in childhood. The ACE Index can inform allocation of resources for prevention and mitigation of ACEs.

## Introduction

In health policy and practice, there is an increasing focus on adversity in childhood. Trauma in early life is associated with health and social problems throughout life, including a wide range of mental and physical illnesses, suicide, homelessness, drug and alcohol use and incarceration.^1–3^ ‘Adverse childhood experiences’ (ACEs) have been defined as ‘potentially traumatic events or chronic stressors that occur before the age of 18 and are uncontrollable to the child’.^4^ Exposure to ACEs has commonly been operationalized in research as a count of 10 experiences: domestic violence; parental separation/divorce; having a parent with a mental health condition; being the victim of abuse (physical, sexual and/or emotional); being the victim of physical or emotional neglect; having a member of the household in prison; and growing up in a household where adults use drugs or alcohol harmfully.

There is now consistent evidence of a graded relationship between ACEs and poor health and social outcomes,^1^^,^^2^ but there are limitations to the literature. Most studies use data from surveys of adults that ask participants to recall ACEs. The validity and biases in these data are difficult to assess, and associations found in these studies may not be generalizable to children today. Studies tend to overlook societal or structural determinants of ACEs such as poverty. This is reflected in current interventions, which are based on a ‘deficit’ model of the family, and particularly mothers, and focus on the quality of parental care.^5^ Families are considered both the cause and the solution to ACEs, while contextual and community factors receive less attention.^6^ Although interventions designed to improve parenting may be effective, they are unlikely to address social inequality in ACEs.

Studies consistently show that adverse childhood experiences are more common among people who reported low socioeconomic position in childhood.^7^^,^^8^ A small number of studies, including survey data of adults in England and Wales,^9^ show that individuals with high ACE scores are more likely to live in deprived areas. The direction of causation is difficult to evaluate because these data are cross-sectional and participants are adults who may have moved since childhood.

An analysis of the geographical patterns of contemporary ACEs and the association with deprivation may provide evidence of the structural causes of ACEs. It may also help with prioritization of resources for prevention and amelioration of ACEs and provide a starting point for local public health teams that want to explore the occurrence of ACEs in their areas in more detail. We aimed to (i) construct a population-level ‘ACE Index’ using publicly available administrative data that identifies recent ACE events (such as police reports of child abuse) and (ii) explore associations between local characteristics and the frequency of ACEs.

## Methods

### Study design

We used ecological data related to the rate ACEs at local authority level in England to construct a composite ‘ACE Index’ and then conducted a cross-sectional ecological analysis of associations with local characteristics such as deprivation and population density.

### Data sources: indicators of adverse childhood experiences

We reviewed publicly available administrative datasets that include: (i) a number of events relevant to existing ACE constructs, such as physical abuse of children; (ii) counts of events at local authority level or smaller; and (iii) data for events occurring in 2010 or later.

Existing ACE constructs often divide experiences into maltreatment and household adversity,^1^ and we grouped indicators into these two domains. We also included a third domain, ‘local context’, for indicators that did not relate directly to a number of ACE events but may provide a proxy (such as suicides and arrests related to drugs, violence and knife crime).

We included the most recent data before 2018 with up to 5 years where multiple years of data were available. We used ONS mid-year population estimates of residents aged 0–18 to calculate rates, unless the data source included a relevant denominator.

**Fig. 1:**
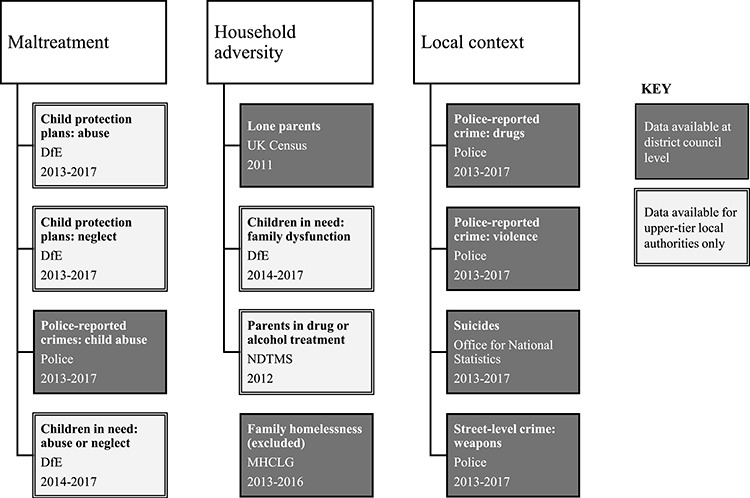
Data sources for ACE indicators in the three domains. DfE = Department for Education (a UK government department). NDTMS = National Drug Treatment Monitoring System (a central database of minimum data sets collected by drug and alcohol services in England). MHCLG = Ministry for Housing, Communities and Local Government (a UK government department).

The indicators and sources that we selected are shown in Fig. 1. Full details of each indicator, including the source and denominator used to calculate rates, are given in Supplementary Information.

### Data sources: health and social effects of ACEs

As well as ACE events, we identified administrative datasets that provide a measure of potential health and social effects of ACEs. We used these data to help design and validate the index, on the assumption that ACEs are correlated with these outcomes. Although ACEs are associated with many health problems throughout life, we focused on outcomes in young people to allow plausible ecological comparisons with the rate of ACE events. We also focused on outcomes that have conceivably high attributable fractions for ACE exposures. For example, we included school exclusions but not stroke, because stroke typically occurs later in life and many strokes occur in people who have not had an adverse childhood (i.e. a low attributable fraction). The outcomes we identified were under 18 conceptions; school exclusions; hospital admissions due to self-harm in 10–24-year olds; hospital admissions due to substance misuse in patients aged under 18; hospital admissions due to alcohol in patients aged under 18; first remands; pupils on the special educational needs register related to social, emotional and mental health problems; school absences at primary level; school absences at secondary level; homelessness at ages 16–24; not in education, training or employment (NEET) at ages 16–17; and school readiness at age 5. As with the ACE indicators, we calculated the rate of outcomes using population denominators. Full details and sources are listed in Supplementary Information.

### Data sources: local characteristics

We characterized local authorities according to deprivation, local inequality and population density. We used the Income Deprivation Affecting Children Index (IDACI) 2015^10^ to identify associations between local authority level deprivation and the ACE Index. IDACI is the proportion of children aged 0–15 living in income-deprived families and is a supplementary domain of the Index of Multiple Deprivation.^11^ In addition, we measured the deprivation using the seven domains of the Index of Multiple Deprivation: income, health, employment, crime, education, living environment and barriers to local services. We calculated local inequality as the ratio between the mean IDACI scores for the bottom and top quartile of neighbourhoods (Lower Super Output Areas, which are small areas with an average population of 1500 are) within a local authority. This measure ranged from 1.3 to 4.8. Population density was defined as the number of residents per hectare in 2011 (the most recent national census year).

### Imputation of district-level indicators

Local government in England is comprised of 152 ‘unitary’ and ‘county’ councils. County councils cover large, mainly rural areas, and each has a second tier of between 4 and 19 ‘district’ councils. All indicators were available for county and unitary councils, but data relating to child protection plans, ‘children in need’ and the number of parents in drug or alcohol treatment were not available for district councils. We therefore imputed these indicators for district councils by (1) fitting a regression model using data from the 152 county and unitary councils where the dependent variable was the indicator to be imputed and a range of independent variables relating to local socioeconomic conditions; (2) predicting the indicator for district councils; and (3) rescaling the predicted values so the total for districts within each country equals the actual value for the relevant county council. These procedures are described in more detail in Supplementary Information.

### Calculation of the ACE Index

We first calculated the ACE Index for unitary and county councils, excluding the City of London and the Isles of Scilly because they are small and unusual areas and often had missing data. Following the methods used to combine indicators in the Index of Multiple Deprivation,^10^ we aggregated indicators into domain scores and then aggregated domain scores into the overall index.

#### Domain scores

We calculated domain scores by taking the mean of z-scores (the difference between the local authority score and the mean, divided by the standard deviation) across each indicator. We used z-scores because indicators had different magnitudes and averaging untransformed rates would prioritize more common (but possibly less serious) events. We used correlations with health and social outcomes as a sense-check of validity of indicators. This led us to exclude family homelessness because it was negatively correlated with most outcomes (correlations are shown in Supplementary Information).

#### Aggregating domain scores into the ACE Index

We considered two stages when combining the domain scores into the index: transformation and weighting. At both stages we used correlations with ACE outcomes as a guide.

We considered three potential transformations of domains: a z-score, rank and the exponential transformation used in the Index of Multiple Deprivation. We calculated candidate ACE Indices as the unweighted mean of the domains under each transformation and then calculated the correlation coefficient between candidate indices and the outcome measures. The rank transformation gave the strongest correlation for 7/11 outcomes, and we therefore transformed local authority domain scores by ranking them.

To determine domain weighting, we identified an ‘optimal’ weighting by testing all combinations of weights between 0.1 and 0.8 (at intervals of 0.01) and selecting the combination where the weighted mean had the strongest correlation with outcomes. Although some of the optimized weights were very unequal (for example, weights of maltreatment = 0.67, household adversity = 0.10 and context = 0.23 produced the strongest correlation with absences from secondary school), in most cases the weights only produced a modest improvement in the correlation (for example, from an increase in R^2^ from 0.42 to 0.48 for absences from secondary school). We therefore used an unweighted mean.

#### Reporting the ACE Index

We presented the ACE Index for local authorities on scatter plots against local characteristics. To further explore geographical associations with ACEs, we fit a linear regression model using ecological data at local authority level with the ACE Index as the outcome. Independent variables were the rank of child poverty (we did not include other Index of Multiple Deprivation domains because they were collinear), population density (with a log transformation due to left skew), local inequality and the geographical region.

Analysis was conducted using R version 3.6.1.

## Results

We developed a composite ACE Index from 11 indicators, each representing a population rate of ACE events, grouped into three domains. The indicator rates varied widely across local authorities. For example, the rate of police-recorded child abuse crimes ranged from 2.8 per 1000 child years in Wokingham (South East England) to 13.2 in Blackpool (North West England). Histograms of the rate of indicators are provided in Supplementary Information.

The ACE Index at local authority level is shown on a map of England in Supplementary Information. The highest ACE rankings are in London, northern cities, the north-east and coastal towns in the South East.

**Fig. 2:**
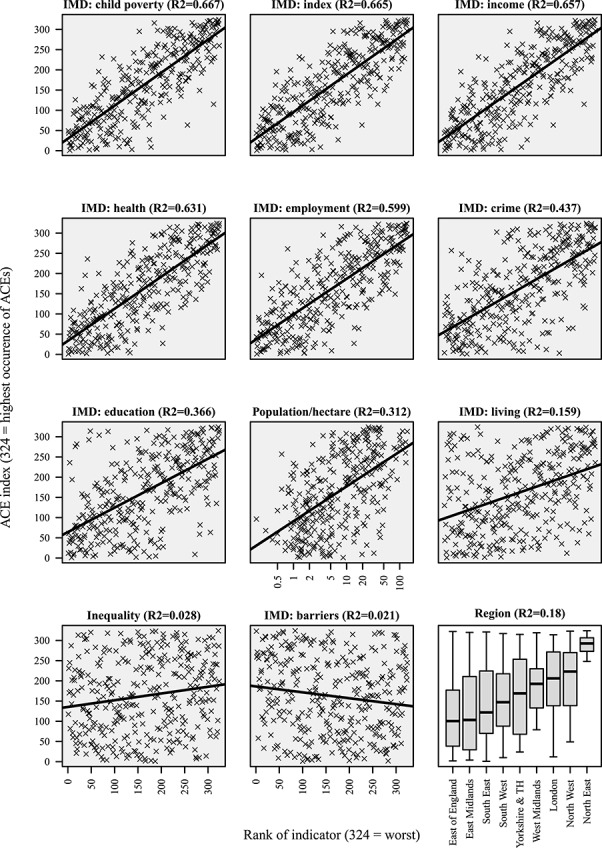
Scatterplot of ACE Index compared to local characteristics.

In bivariate scatterplots, child poverty and low income were the local characteristics most strongly associated with the ACE Index (Fig. 2). Local health, employment, crime and population density were also strongly associated with the ACE Index. Associations with deprivation related to education and living conditions were weaker. We did not observe any association between the ACE Index and local inequality or ‘barriers to services’.

**Fig. 3:**
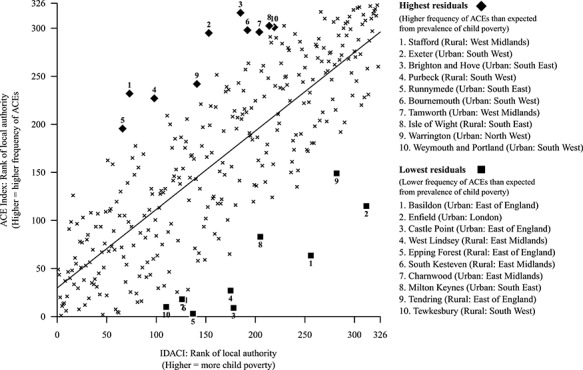
Scatterplot of child poverty against the ACE Index at local authority level. Local authorities that are furthest from the line of best fit are highlighted. ‘Rural’ areas are those in the bottom half of population density values; ‘urban’ areas are those in the top half.

Fig. 3 shows the ACE Index compared to child poverty, highlighting local authorities that are furthest from a line of best fit derived from linear regression.

The ecological regression (Table 1) showed that child poverty and higher population density are both strongly associated with a higher rank on the ACE Index. The association between child poverty and the ACE Index is not explained by population density or region. The association between population density is attenuated, but not entirely, by deprivation (urban areas in England are on average more deprived), and an increase of one standard deviation in population density was independently associated with an increase of 15/324 ranks. Local authorities in London, which have the highest rate of child poverty in the country, on average had the third highest ACE Index out of the nine regions in England. However, after adjustment for child poverty and population density, London had the lowest expected ACE Index. We observed little association between local inequality and the ACE Index.

**Table 1:** Results of linear regression for local authority characteristics on the ACE Index (95% CIs)

	*Unadjusted*	*Fully adjusted*
**Continuous variables**		
IDACI (z-score of rank)	76.50 (70.57, 82.43) *P* < 0.001	70.70 (62.86, 78.54) *P* < 0.001
Density (z-score of log residents per hectare)	52.42 (43.83, 61.01) *P* < 0.001	15.49 (6.40, 24.58) *P* < 0.001
Local inequality (z-score)	15.59 (5.46, 25.72) *P* = 0.003	−3.04 (−9.52, 3.43) *P* = 0.356
**Region**		
East of England (reference)	*116.23 (91.63, 140.84)* ^*^	*132.13 (117.60–146.65)* ^*^
East Midlands	9.75 (−26.53, 46.04) *P* = 0.597	5.07 (−15.74, 25.87) *P* = 0.632
South East	27.68 (−4.42, 59.77) *P* = 0.091	50.11 (31.09, 69.12) *P* < 0.001
South West	37.65 (0.30, 75.01) *P* = 0.048	52.47 (31.06, 73.88) *P* < 0.001
Yorkshire and the Humber	43.69 (−0.58, 87.97) *P* = 0.053	24.28 (−1.78, 50.34) *P* = 0.068
West Midlands	70.32 (30.90, 109.73) *P* < 0.001	49.38 (26.78, 71.99) *P* < 0.001
London	81.27 (42.61, 119.93) *P* < 0.001	−20.89 (−47.46, 5.68) *P* = 0.123
North West	94.69 (58.15, 131.23) *P* < 0.001	50.81 (29.12, 72.50) *P* < 0.001
North-east	172.47 (117.92, 227.03) *P* < 0.001	78.78 (46.01, 111.54) *P* < 0.001

Effect sizes represent a number of ranks in the ACE Index (out of 324).

IDACI = Income Deprivation Affecting Children Index

^*^Coefficients for East of England are the intercept values (i.e. the average rank for local authorities in the East of England).

## Discussion

### Main findings of this study

We found that the frequency of ACEs measured using administrative data varied widely across England and was highest in areas with high rates of child poverty. Higher rates of ACEs were also predicted by population density and region, with local authorities in the north-east in particular having a higher-than-expected rate.

### What is already known on this topic

Previous research based on surveys of adults has found a graded relationship between the number of adverse experiences in childhood and risk of health and social problems throughout life.^1^^,^^9^^,^^12^^,^^13^ Research has also shown that adults who had adverse childhoods are more likely to live in more deprived areas.^9^ Authors of these studies have suggested that ACEs cause poverty later in life, which would explain this association. Another recent study uses birth cohort data to show that ACEs are over 10 times more likely to occur in the poorest 20% of the population compared to the richest 20%.^14^

### What this study adds

Our results show that local areas with high rates of child poverty also have a high frequency of ACEs. This suggests that the known association between deprivation and ACEs among adults is unlikely to be explained by recall bias or selective migration but is because children growing up in these areas have higher risk of adverse experiences. It also provides evidence for a process in which deprivation increases the risk of adverse experiences in childhood. Deprivation and economic downturns are associated with social problems including drug and alcohol dependence,^15^ involvement in crime, mental health problems,^16^^,^^17^ homelessness, unemployment and debt. These problems may increase household adversity and affect parenting, put children and young people more at risk of involvement in crime and gangs and damage community cohesion and resilience. Our data is ecological, and it is possible that the association between deprivation and the frequency of ACEs does not hold at a household or individual level. However, we could not identify plausible mechanisms that would otherwise explain the association. The strength of the association and the ‘upstream’ nature of deprivation (meaning that reverse causality is unlikely) support a causal relationship.

In one US study, changes in minimum wages were used to explore the impact of increased family income on child abuse and neglect.^18^ A $1 increase in the minimum wage was associated with a 10% decline in neglect reports. These results provide evidence that improvements in socioeconomic status can lead to a decrease in childhood adversity.

The regression analysis identified additional sources of variation beyond deprivation. The north-east, for example, had a substantially higher rate of ACEs than could be explained by child poverty. The north-east also has a high rate of other social problems, including the highest rate in England of drug-related deaths,^19^ alcohol-specific deaths,^20^ unemployment^21^ and suicides.^22^ These problems may cluster in families or communities and lead to a higher risk of ACEs. In addition, certain other areas, including the south coast towns of Brighton and Hove, Bournemouth and the Isle of Wight, had higher ACE Indices than expected from their level of child poverty (Fig. 3).

Cities were generally ranked higher than rural areas, reflected in the independent association between the ACE index and population density. Urban areas with high ACE indices do not always have high average deprivation. For example, the London Borough of Islington, which had the third highest median gross weekly wage in January 2019 at £690,^23^ is ranked similarly on the ACE Index to Hull, with the 13th lowest weekly wage at £380. Rural areas generally rank lower on the ACE index, with some exceptions, such as counties in the north-east and Cornwall. A more detailed analysis of the contextual predictors of ACEs may consider additional factors to explain this variation, such as economic history, the presence of local criminal activities and availability and quality of specific public services.

The process of constructing the index uncovered gaps in data. For example, studies of ACEs typically ask adults if they witnessed domestic abuse as a child, but we were unable to find data showing the number times domestic abuse was reported locally, and also ask about parents’ mental health problems, for which we were also unable to find relevant data.

The results are not intended to be generalizable to other countries.

### Limitations of this study

There are unknown biases in the collection of administrative data. For example, services that contributed data to our index are more likely to have offices in cities, which may mean that adverse events are more likely to be detected and recorded if they occur in cities. This may contribute to the association that we observed between population density and the ACE Index. Arguably, a higher ACE Index may reflect more effective local services and a higher likelihood of intervention when problems occur. However, the correlation between the ACE Index and outcomes among people aged under 18, including the rate of first remands or school exclusions (see Supplementary Information), suggests that the index primarily measures the occurrence of ACEs in the population rather than the quality of local services.

The common approach to quantifying exposure to ACEs, based on 10 experiences,^4^ has predictive validity (meaning it is associated with expected outcomes) but has been criticized. In particular, there is no clear rationale for the list of ACEs. Lone parenting is common and may not be adverse, and in some cases parental separation may be protective. Longitudinal data show that it is not parental separation that is detrimental to child well-being but its interaction with poverty.^24^ Additional experiences such as bullying, bereavement, gang membership, being a victim of violent crime and witnessing community violence have also been included in some studies of ACEs. These extended definitions can have stronger associations with health and social outcomes in later life.^25^ We did not aim to critically assess which events should be considered ‘ACEs’, and the index may be improved by inclusion of additional or different data.

We did not attempt to calculate the ACE Index for areas that are smaller than local authorities. This was because (i) most of our data is not available for small areas and (ii) the rate of most events would be too low to observe meaningful variation. However, local authorities are often diverse, and the risk of ACEs may vary substantially within them.

## Conclusion

The rate of adverse childhood experiences in England is strongly associated with child poverty and provides evidence for a process in which deprivation increases the risk of adverse experiences in childhood. There are substantial additional sources of variation that may warrant further research. The ACE Index could be used to inform allocation of resources for services that prevent and mitigate child adversity and to monitor changes over time.

## Supplementary Material

ACE_index_supplementary_fdz158

full_results_fdz158

## Study approval

All data used in this study is publicly available, and no ethical consideration or other approval was required.

## Notes

**Dan Lewer**, Doctoral Research Fellow

**Emma King**, Public Health Specialty Registrar

**Glen Bramley**, Professor of Urban Studies

**Suzanne Fitzpatrick**, Professor of Housing and Social Policy

**Morag Treanor**, Professor of Child and Family Inequalities

**Nick Maguire**, Associate Professor

**Miriam Bullock**, Public Health Specialty Registrar

**Andrew Hayward**, Professor of Infectious Disease Epidemiology and Inclusion Health Research

**Al Story**, Clinical Lead, Find & Treat
