# Erratum: The ACE Index: mapping childhood adversity in the UK

**DOI:** 10.1093/pubmed/fdaa026

**Published:** 2020-02-20

**Authors:** Dan Lewer, Emma King, Glen Bramley, Suzanne Fitzpatrick, Morag Treanor, Nick Maguire, Miriam Bullock, Andrew Hayward, Al Story

In the original article, a section was missed from the Funding information. This has now been added. In addition some changes have been made to the supplementary material:

- Corrections to the map of the ACE Index, including some changes to the local authority rankings and inclusion of a high-definition figure
- Corrections to the correlations shown in section 2
- Corrections to some denominators shown in sections 4 and 5

The Publisher apologizes for the errors.

