# Supplementary material for: The ACE Index: mapping childhood adversity in England
Source: J Public Health (Oxf). 2019 Dec 27;42(4):e487–95. doi: 10.1093/pubmed/fdz158 (PMC7685852; doi:10.1093/pubmed/fdz158)
Supplement: ACE_index_supplementary_fdz158 [file ace_index_supplementary_fdz158.pdf]

Supplementary Information

1. Map of the ACE Index
2. Associations between the ACE index, its constituent domains and indicators, and the social and health effects of ACEs
3. Population rate of ACE indicators
4. Sources for ACE indicators, and denominators
5. Sources for the health and social effects of ACEs, and denominators
6. Imputation of district-level rates of child protection plans, children-in-need events and parents in drug and alcohol treatment
7. References for supplementary information

1 Map of the ACE Index

Rank of ACE Index by local authority. Higher rank indicates that ACEs are more frequent.

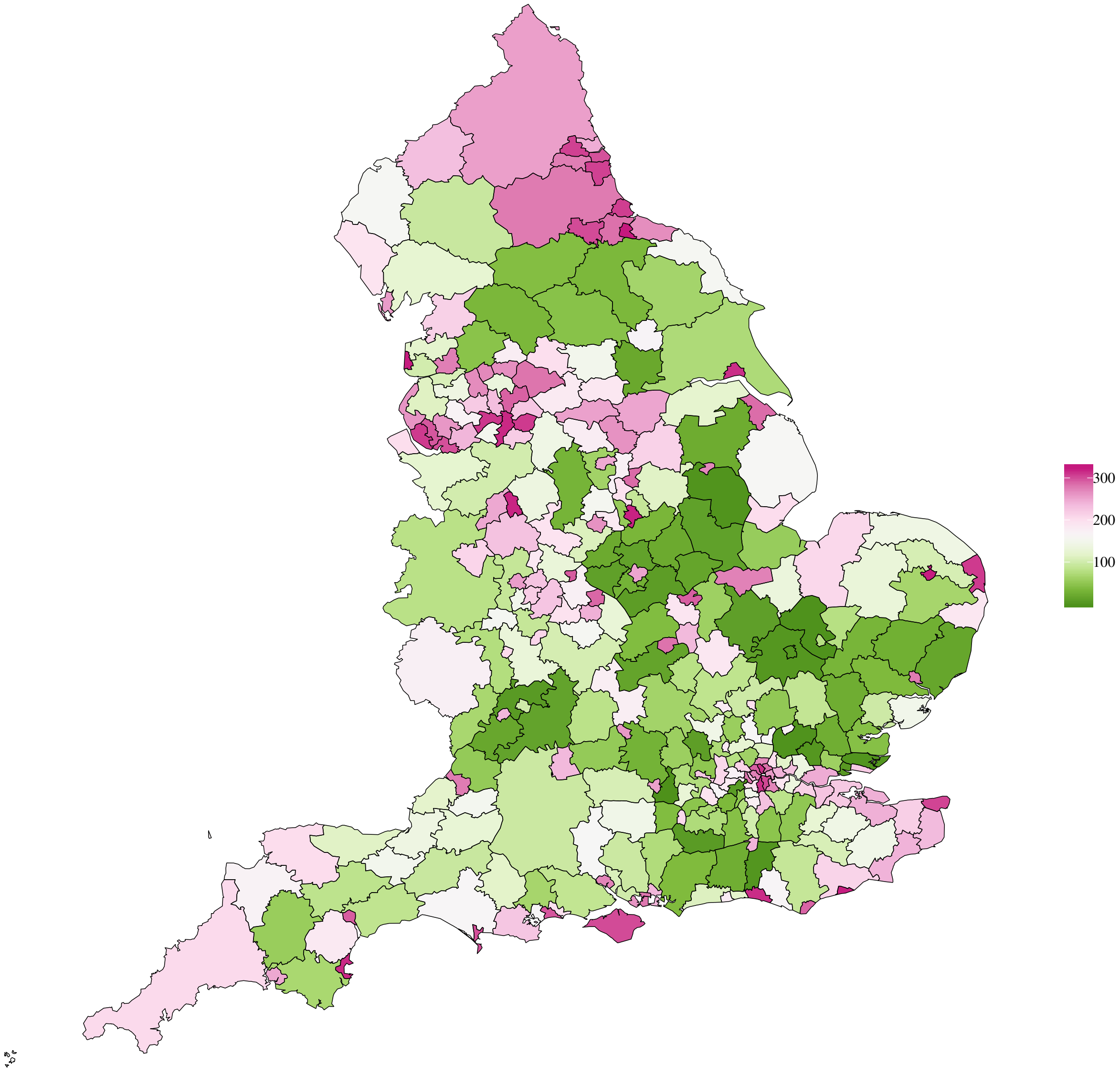

2 Associations between the ACE index, its constituent domains and indicators, and the social and health effects of ACEs

The figure below shows ecological correlations between the ACE Index and the potential health and social effects of ACEs. We used these correlations to help us design the ACE Index, including whether any indicators should be excluded and how domains should be transformed and weighted. We excluded family homelessness because it was negatively associated with many of the variables. This may be because accommodation is more expensive in wealthy areas that otherwise have lower rates of ACEs. At an individual family level, family homelessness is likely to be positively associated with ACEs, so this may reflect an ‘ecological paradox’. The meaningfulness of correlations for the domain scores and the ACE Index are limited by the fact that we used these variables to inform the design of the ACE Index, so the correlations are optimised.

Figure: Pearson’s correlation coefficient between rates of ACEs and the health and social effects of ACEs, for local authorities in England

	Under 18 conceptions	School exclusions	Admissions for self harm (age 10–24)	First remands	SEN register (SEM)	Primary school absences	Secondary school absences	Homelessness (age 16–24)	Admissions for substance use (under 18)	Admissions for alcohol (under 18)	NEET	School readiness
**ACE index**	71	32	20	62	42	61	43	5	39	40	1	46
**Maltreatment domain**	79	38	36	52	34	51	49	−9	46	49	2	48
CPP: abuse	50	18	27	30	26	42	31	−10	32	38	0	39
CPP: neglect	60	30	34	29	21	24	39	−19	38	50	−2	26
Police: abuse	62	44	25	57	32	40	43	3	28	29	3	35
CIN: Abuse or neglect	67	24	24	41	23	46	33	−2	40	29	5	44
**Household domain**	50	22	11	35	31	47	32	7	34	27	−2	29
CIN: family	17	16	12	7	11	14	18	−7	21	29	−7	1
Family homelessness	−10	−20	−52	29	15	17	−29	82	−36	−50	−7	−15
Lone parents	51	9	−21	60	32	59	14	35	12	−6	2	28
Parents in drug/alcohol treatment	35	20	31	5	20	24	32	−12	37	33	−1	30
**Context domain**	44	31	12	56	43	50	38	7	23	26	4	34
Police: drugs	1	−1	−29	39	28	31	2	21	−12	−13	−5	10
Police: violence	46	34	15	54	41	42	34	8	25	22	1	29
Suicide	43	37	62	12	24	22	55	−33	54	66	−1	25
Police: weapons	38	19	−14	55	29	47	16	24	−2	−4	18	34

3 Population rate of ACE indicators

Histograms of frequency of individual ACE indicators by local authority. X-axes show the rate of events per 1000 person-years. 'Lone parents' is given as a prevalence of lone parents per 1000 households with dependent children

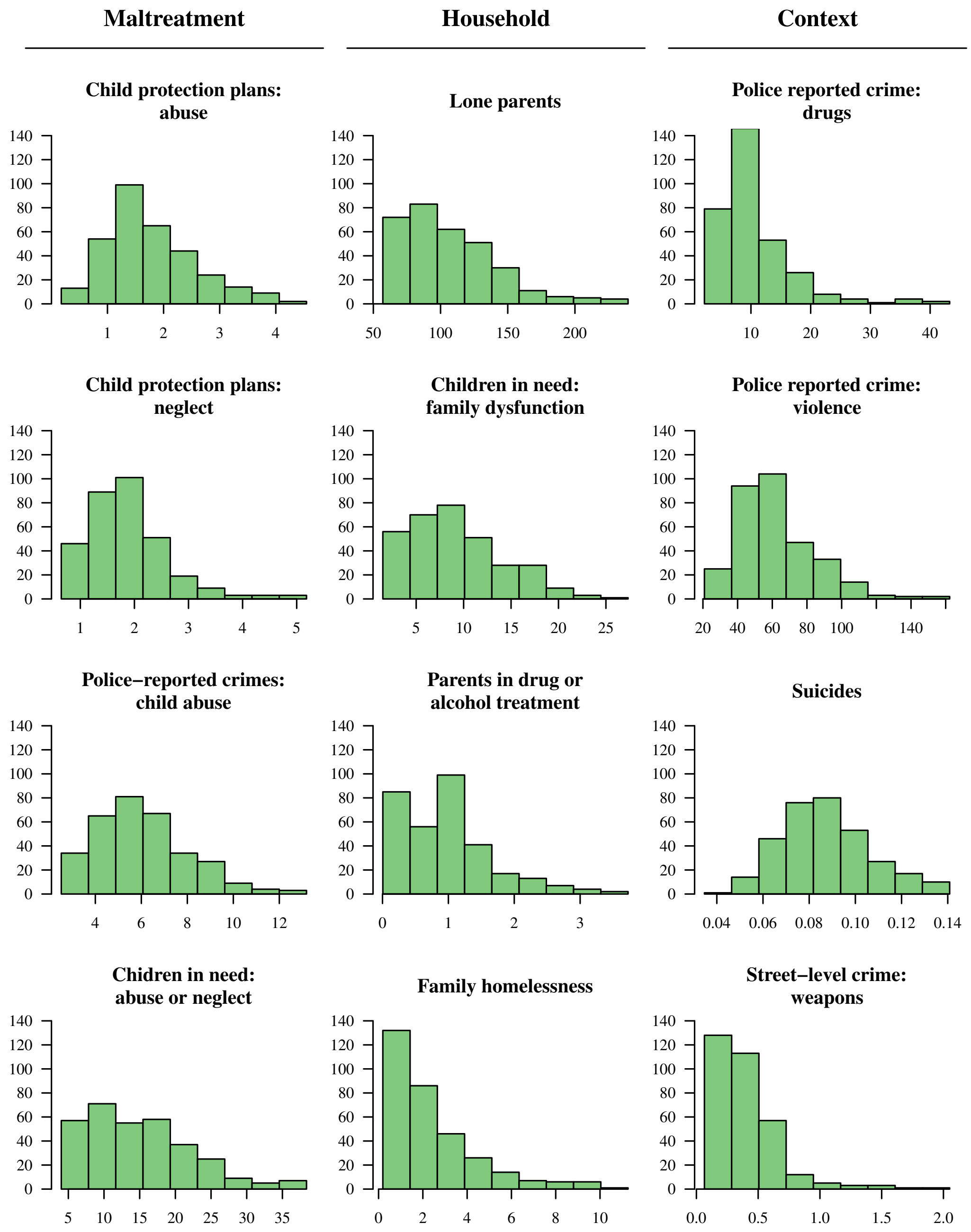

4 Sources for ACE indicators, and denominators

Indicator	Source	Smallest area of data availability	Years	Count of events	Denominator age range	Denominator (person-years)
Maltreatment domain						
Police recorded crime: abuse	Police[1]	Community Safety Partnership	2013-2017	476,890	0-18	61,689,287
CPP: neglect	Department for Education[2]	County or unitary authority	2013-2017	116,766	0-18	61,689,287
CPP: abuse	Department for Education[2]	County or unitary authority	2013-2017	117,767	0-18	61,689,287
CIN: abuse and neglect	Department for Education[2]	County or unitary authority	2014-2017	811,692	0-18	49,532,626
Household adversity domain						
CIN: household dysfunction	Department for Education[2]	County or unitary authority	2014-2017	450,142	0-18	49,532,626
Family homelessness	Ministry of Housing Communities and Local Government[3]	District council	2013-2016	147,034	0-18	49,177,197
Lone parents	Census 2011[4]	District council	2011	1,671,396	Households*	14,448,646
Parents in drug/alcohol treatment	National Drug Treatment Monitoring System[5]	County or unitary authority	2011/12	11,830	0-15*	10,030,300
Context domain						
Police recorded crime: drugs	Police[1]	Community Safety Partnership	2013-2017	852,217	0-18	61,689,287
Police recorded crime: violence	Police[1]	Community Safety Partnership	2013-2017	4,749,641	0-18	61,689,287
Street-level crime: possession of weapons	Police[1]	Latitude and Longitude of crime	2013-2017	120,319	0-90	273,856,259
Suicides	Office for National Statistics[6]	District council	2013-2017	23,455	0-90	273,856,259

* Denominator is provided with the dataset

5 Sources for the health and social effects of ACEs, and denominators

Variable	Source	Years	Count of events	Denominator age range	Denominator (person-years)
Under 18 conceptions	Office for National Statistics[7]	2013-2016	104,943	Female age 13-17	7,473,735
School exclusions	Department for Education[8]	2011/12-2016-17	1,900,513	School pupils*	46,601,942
Admissions for self-harm (age 10-24)	Public Health England Fingertips[5]	2012-2016	197,229	Oct-24	49,176,498
First time entrants to young justice system	Public Health England Fingertips[5]	2013-2016	76,908	10-17*	19,615,416
SEN register (SEM)	Department for Education[9]	2013-2016	733,794	School pupils*	32,444,871
Primary school absences	Department for Education[10]	2014/15-2016/17	954,294	School pupils*	11,462,677
Secondary school absences	Department for Education[10]	2014/15-2016/17	1,152,521	School pupils*	8,553,412
Homelessness (age 16-24)	Ministry of Housing Communities and Local Government[3]	2013-2017	65,239	16-24	30,825,853
Hospital admissions due to substance misuse (15-24)	Public Health England Fingertips[5]	2011/12-2016/17	34,400	15-24*	40,085,143
Hospital admissions due to alcohol (under 18)	Public Health England Fingertips[5]	2011/12-2016/17	25,882	0-18*	67,960,650
Not in employment, education or training	Department for Education[11]	2016	415,350	16-17*	1,155,390
School readiness	Public Health England Fingertips[5]	2012/13-2017/17	1,185,193	5*	3,271,224

* Denominator is provided with the dataset

6 Imputation of district-level rates of child protection plans, children-in-need events and parents in drug and alcohol treatment

Local authorities in England can be classified into three groups: (1) county councils, which cover 27 large, mainly rural areas, and provide one tier of services; (2) district councils, which are smaller sub-areas within the county councils; and provide a second tier of services; and (3) unitary authorities, which cover mainly urban areas and provide both tiers of services. Within each county there are 4 to 19 districts. One issue in developing the ACE index was that five indicators related to services provided by county or unitary authorities, and data were not available at the smaller district council level. For example, children's social care is provided by county councils, and therefore the data on child protection plans and 'children in need' are only available at county or unitary level. We therefore imputed these indicators for district councils using the following approach:

1. We fit a poisson regression model of 150 county and unitary authorities, with the count of events (e.g. child protection plans related to abuse) as the dependent variable, a number of independent variables that are available for all types of local authority, and offset by the log population (or person-years if the counts were taken over more than one year). As independent variables, we used the population density, proportion of children living in income deprived households (the IDACI measure of the Index of Multiple Deprivation 2015), the rate of police-recorded crimes related to abuse, the rate of all street-level crimes (which tend to relate to crimes such as violence, theft and shoplifting rather than abuse), the proportion of families with lone parents, and the rate of teenage conceptions.
2. We used the model to predict the count of events for district councils, based on the independent variables and the population (or person-years) for the district council.
3. Within each county, we re-scaled the counts for districts so the predicted total across the districts equalled the actual total for the county. We did this by calculating a ratio between the predicted total and the actual total, and applying it to the predicted district values.
4. We calculated a rate for the districts based on the re-scaled predicted count and the district population (or person-years).

We repeated this process across each of the five indicators. The plots below show the county-level values and the imputed values. The charts include unitary as well as district and county councils. For many ACE indicators, unitary councils have the highest rates as they often include poor urban areas. These areas form the higher rates at the top-right of the plots with no corresponding district councils. The imputation process added substantial variation, but the dominant determinant of imputed values remained the rate in the local county council.

Figure: Observed rates for county and unitary authorities compared to imputed rates for district councils

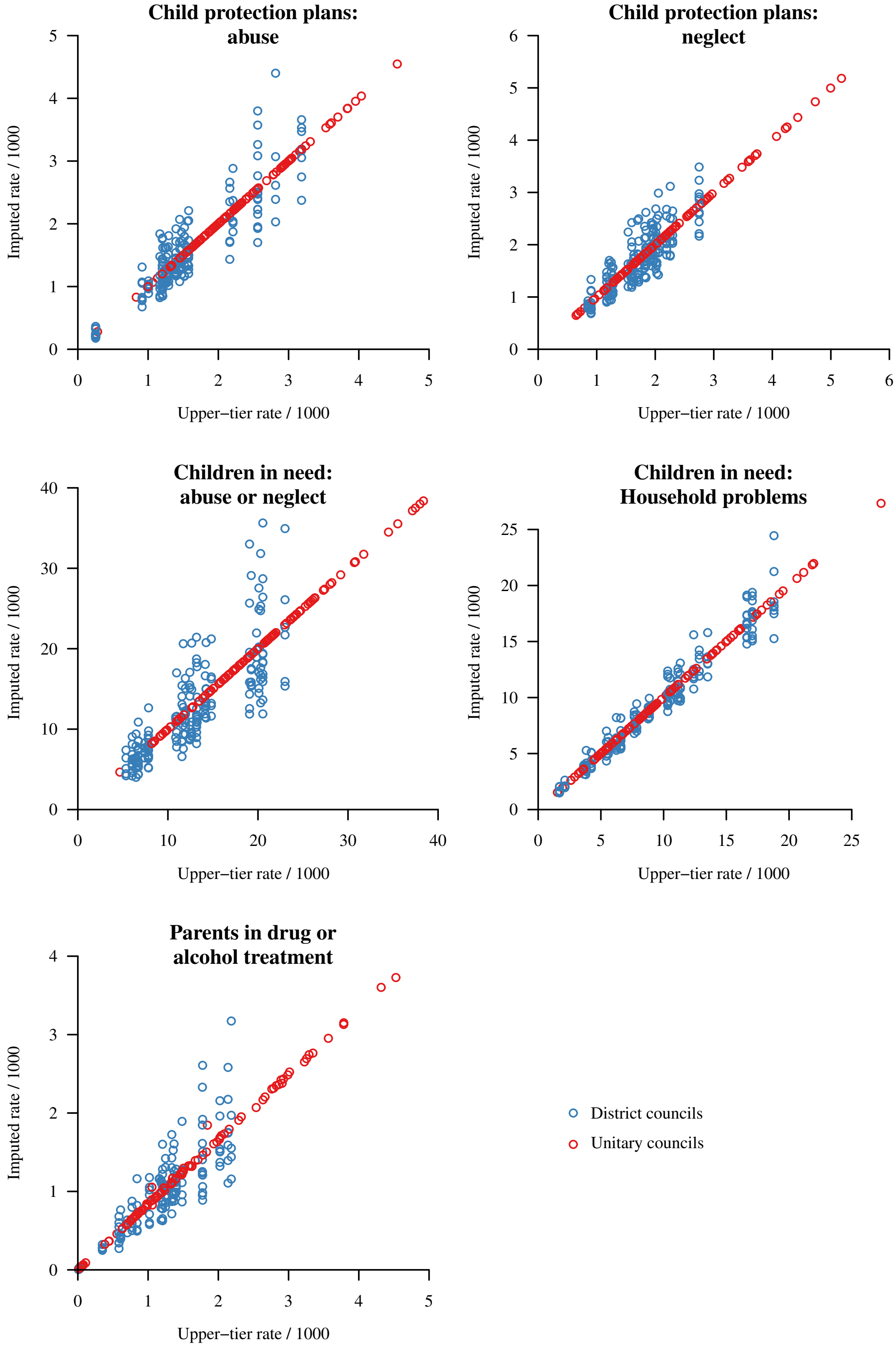

7 References for supplementary information

1. Home Office. Police Open Data. 2019. https://data.police.uk/data/ (accessed Sept 25, 2019).
2. Department for Education. Statistics: children in need and child protection. 2018. https://www.gov.uk/government/collections/statistics-children-in-need (accessed Sept 25, 2019).
3. Ministry of Housing, Communities & Local Government. Homelessness Statistics. 2019. https://www.gov.uk/government/collections/homelessness-statistics (accessed Sept 25, 2019).
4. Office for National Statistics. 2011 Census: Key Statistics for England and Wales, March 2011. 2012. https://www.ons.gov.uk/peoplepopulationandcommunity/populationandmigration/populationestimates/bulletins/2011censuskeystatisticsforenglandandwales/2012-12-11 (accessed Sept 25, 2019).
5. Public Health England. Fingertips: Children and Young People's Mental Health and Wellbeing. 2015. https://fingertips.phe.org.uk/cypmh#page/0/gid/1938133090/pat/6/par/E12000004/ati/102/are/E06000015 (accessed Sept 25, 2019).
6. Office for National Statistics. Suicides in England and Wales by local authority. 2019. https://www.ons.gov.uk/peoplepopulationandcommunity/birthsdeathsandmarriages/deaths/datasets/suicidesbylocalauthority (accessed Sept 25, 2019).
7. Office for National Statistics. Conceptions in England and Wales: 2017. 2019. https://www.ons.gov.uk/peoplepopulationandcommunity/birthsdeathsandmarriages/conceptionandfertilityrates/bulletins/conceptionstatistics/2017 (accessed Sept 25, 2019).
8. Department for Education. Statistics: exclusions. 2019. https://www.gov.uk/government/collections/statistics-exclusions (accessed Sept 25, 2019).
9. Department for Education. Statistics: special educational needs (SEN). 2019. https://www.gov.uk/government/collections/statistics-special-educational-needs-sen (accessed Sept 25, 2019).
10. Department for Education. Statistics: pupil absence. 2019. https://www.gov.uk/government/collections/statistics-pupil-absence (accessed Sept 25, 2019).
11. Department for Education. Statistics: NEET and participation. 2019. https://www.gov.uk/government/collections/statistics-neet (accessed Sept 25, 2019).
